# Robust Deep Active Learning via Distance-Measured Data Mixing and Adversarial Training

**DOI:** 10.3390/e27111159

**Published:** 2025-11-14

**Authors:** Shinan Song, Xing Wang, Shike Dong, Jingyan Jiang

**Affiliations:** 1 School of Computer Science and Engineering, Changchun University of Technology, Changchun 130012, China; songshinan@ccut.edu.cn (S.S.);; 2 Sendelta International Academy Shenzhen, Shenzhen 518100, China; 3 College of Bigdata and Internet, Shenzhen Technology University, Shenzhen 518118, China

**Keywords:** active learning, data selection, robustness, uncertainty estimation

## Abstract

Accurate uncertainty estimation in unlabeled data represents a fundamental challenge in active learning. Traditional deep active learning approaches suffer from a critical limitation: uncertainty-based selection strategies tend to concentrate excessively around noisy decision boundaries, while diversity-based methods may miss samples that are crucial for decision-making. This over-reliance on confidence metrics when employing deep neural networks as backbone architectures often results in suboptimal data selection. We introduce Distance-Measured Data Mixing (DM2), a novel framework that estimates sample uncertainty through distance-weighted data mixing to capture inter-sample relationships and the underlying data manifold structure. This approach enables informative sample selection across the entire data distribution while maintaining focus on near-boundary regions without overfitting to the most ambiguous instances. To address noise and instability issues inherent in boundary regions, we propose a boundary-aware feature fusion mechanism integrated with fast gradient adversarial training. This technique generates adversarial counterparts of selected near-boundary samples and trains them jointly with the original instances, thereby enhancing model robustness and generalization capabilities under complex or imbalanced data conditions. Comprehensive experiments across diverse tasks, model architectures, and data modalities demonstrate that our approach consistently surpasses strong uncertainty-based and diversity-based baselines while significantly reducing the number of labeled samples required for effective learning.

## 1. Introduction

Deep neural networks typically require extensive labeled datasets for effective training, making data annotation a slow, expensive, and complex process [1]. Active learning (AL) addresses this challenge by strategically selecting the most informative samples from an unlabeled pool for annotation, thereby reducing the overall labeling burden [2]. A prevalent approach prioritizes samples with low-confidence predictions, as these high-uncertainty instances are empirically proven to provide valuable information for model improvement [3]. However, strategies that rely exclusively on uncertainty estimation often concentrate sample selection within narrow regions of the feature space, resulting in inadequate coverage of the overall data distribution and potential amplification of label noise [4]. Incorporating diversity considerations into the selection process can mitigate these issues by ensuring broader distributional coverage and capturing richer information content across the data manifold.

Recent active learning research has therefore pursued two complementary objectives: uncertainty estimation and diversity promotion. Uncertainty-based methods such as Least Confidence [3,5,6] prioritize samples exhibiting minimal predictive confidence. Deep Bayesian techniques further enhance uncertainty estimation by utilizing posterior predictive distributions to refine entropy-based and mutual-information-based selection criteria  [7,8,9,10,11]. Alternative approaches employ auxiliary models to improve uncertainty estimation or guide the selection process  [12,13,14,15,16,17]. Complementing these uncertainty-focused strategies, diversity-oriented methods such as VAAL [18] explore varied regions within the latent space, while BADGE [19] selects points via k-means++ in gradient-embedding space to jointly encourage diversity and gradient-driven uncertainty. Mixing-based active learners like Alpha-Mix also synthesize inputs, but they score and select anchors before mixing and do not explicitly target boundary sensitivity within the AL loop. A brief comparison of pipeline choices highlights these differences: while BADGE relies on gradient embeddings for selection diversity and Alpha-Mix leverages mixing as post-selection augmentation, DM2 couples metric-driven neighbor matching with mixed-sample scoring and adversarial perturbations, thereby unifying diversity, calibrated uncertainty near the boundary, and robustness within a single AL loop. Despite these advances, a fundamental trade-off remains: uncertainty-centric querying can oversample noisy boundary points, whereas diversity-only selection may overlook the most decision-relevant instances in complex, imbalanced, or noisy data—motivating designs like DM2 that explicitly integrate boundary awareness with coverage.

**Figure 1:**
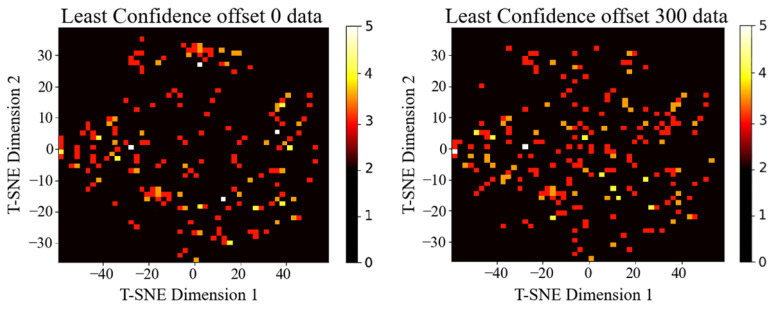
The left side of the figure illustrates a heat map of the data distribution for the Least Confidence method, achieving 83.5% accuracy. The right side depicts the data distribution when offsetting 300 data points, resulting in 84.11% accuracy. This comparison highlights the impact of offsetting on sample diversity and model performance.

From a decision boundary perspective, samples with high uncertainty typically reside near class separators, where noise and label ambiguity are most prevalent. Excessive emphasis on such points can propagate labeling errors and compromise training quality. To investigate this phenomenon, we conducted a preliminary study using Least Confidence on CIFAR-10 [20] with MobileNet [21] as the backbone architecture. Following an initial training phase, we selected 1000 samples per iteration based on lowest confidence scores and visualized the selections using t-SNE, comparing direct top-k selection (offset 0) with an offset strategy that skips the top 300 lowest-confidence samples. As illustrated in Figure 1, the offset strategy distributes selections more broadly across the feature space, enhancing both coverage and diversity. This approach achieved 84.11% accuracy compared to 83.5% without offset, demonstrating that strategically shifting away from the most uncertain samples can capture richer information content and improve overall performance.

Motivated by these observations and drawing inspiration from Alpha-Mix [1], which employs tuned mixing strategies to introduce variability while preserving salient features, we propose a distance-measured data mixing framework (DM2) for deep active learning that simultaneously addresses uncertainty estimation, diversity promotion, and robustness enhancement. Our method DM2 introduces three algorithmic novelties: (i) neighbor selection in the representation space using a combined $\ell_{1}+\ell_{2}$ distance to robustly pair anchors with semantically proximate yet distinct neighbors; (ii) scoring on mixed samples rather than on anchors, which directly estimates the informativeness of interpolated boundary cases; (iii) an explicit boundary-aware adversarial augmentation step integrated into each query round to probe model fragility near decision surfaces. Building upon this foundation, we further introduce a boundary-aware feature fusion mechanism via adversarial training: we generate adversarial counterparts for selected near-boundary samples using fast gradient methods and train them jointly with the original instances. This approach enhances generalization capabilities and robustness in complex, noisy environments by stabilizing the learning process around decision boundaries.

Our contributions are summarized as follows:

- We introduce Distance-Measured Data Mixing (DM2) Active Learning, a novel deep active learning framework that estimates sample uncertainty through distance-weighted mixing of data samples. By exploiting inter-sample relationships and distributional structure, this method selects informative instances across the data manifold, including near-boundary regions, thereby enhancing the diversity of queried samples.
- To address noise susceptibility in challenging scenarios, we augment Distance-Measured Data Mixing with adversarial training (DM2-AT). We generate fast gradient adversarial samples for selected near-boundary instances and train them jointly with the original data, improving model robustness and generalization performance under complex data distributions.
- Comprehensive experiments across diverse tasks, model architectures, and data modalities demonstrate that our method achieves superior performance while significantly reducing labeling requirements compared to existing approaches.

## 2. Related Work

Uncertainty-based approaches select the most ambiguous unlabeled samples according to the current model. Since the model is initially trained with a limited dataset, these ambiguous samples provide valuable information for subsequent training rounds. (1) Prominent uncertainty selection methods include Least Confidence [5] and Entropy Sampling [22]. The Margin Sampling method [6] evaluates the difference between the confidence levels of the highest and second-highest prediction classes. BatchBALD [23] selects samples by maximizing the joint information gain of a batch. (2) Bayesian framework approaches focus on model parameters [4,24], often integrating Bayesian belief networks with Monte Carlo sampling [25]. Deep Bayesian approximation methods like MC-Dropout [7] are employed to address the challenge of probabilistic prediction. Query by Committee (QBC) methods facilitate multi-model training [26], while adversarial training methods [10,11,18] provide additional robustness. Furthermore, the variance between predicted probabilities within a set [27] has been proposed as a measure of uncertainty. (3) Model-based active learning trains a separate model for active instance selection. Variational Autoencoders (VAEs) [18] utilize a V-shaped autoencoder to model data distribution. CoreGCN [13] employs Graph Convolutional Networks (GCNs) to represent relationships between examples. LL4AL [14] integrates a lightweight module to learn the prediction error of unlabeled examples, capturing the learning loss in active learning. ProbCover [28] is a novel active learning algorithm designed for low-budget scenarios, aiming to maximize probability coverage. Methods like ISAL and ent-gn [15,16] propose using influence functions [17] to estimate potential model changes, thereby informing training strategies. These techniques often prioritize points near the decision boundary. However, they may overlook valuable data away from the decision boundary by relying solely on predicted class likelihood.

In active learning, diversity refers to selecting representative and varied samples for labeling. Methods often assign confidence scores based on classifier uncertainty and sample diversity [29]. One strategy uses entropy and mutual information within a CRF graphical model for query selection [30]. The state-of-the-art method Coreset [12] focuses on selecting data with diverse representations. BADGE [19] explores the relationship between data diversity and uncertainty using Bayesian methods and clustering. CoreGCN [13] employs graph embeddings for diverse data selection. Hierarchical agglomerative clustering (HAC) [31] distributes uncertain examples across clusters. BatchBALD [23] also considers sample diversity by selecting complementary samples to avoid redundancy. Recent advances utilize parameters from the final neural layer, while Alpha-Mix [1] employs feature mixing with alpha values. Noise Stability [32] introduces a greedy algorithm that adds noise to highlight differences between samples. These methods propose complex paradigms for modeling diversity, enhancing effectiveness but increasing computational complexity and model coupling.

## 3. Distance-Measured Data Mixing

**Figure 2:**
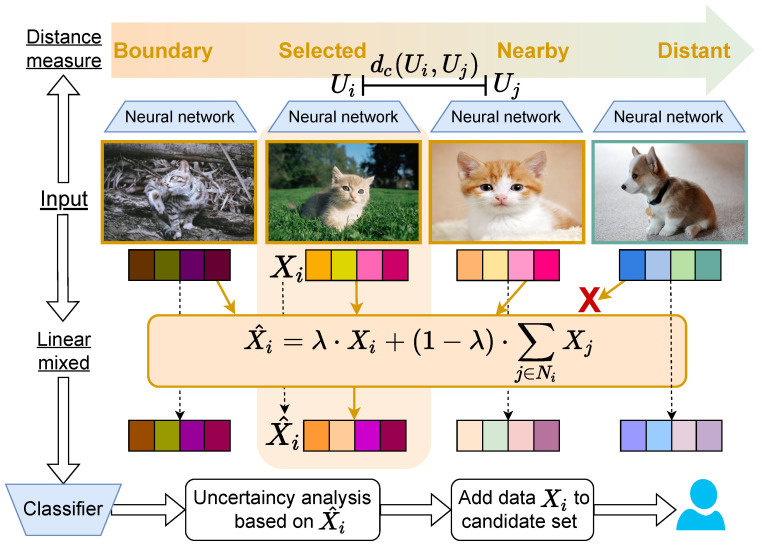
Overview of DM2. Data is input into the model to obtain feature-layer embeddings. We calculate sample similarity distances on these embeddings and select similar samples for linear blending. The model is tested with mixed samples to determine confidence levels. Data with low confidence is identified, and the original data is added to the labeled data pool.

This section presents our theoretical framework for Distance-Measured Data Mixing (DM2), as illustrated in Figure 2. Our approach focuses on selecting samples that effectively balance diversity and uncertainty considerations. The process begins by feeding unlabeled data through the model to extract feature-layer embeddings. We then compute similarity distances between samples within these embedding spaces and select multiple similar instances for linear mixing according to predetermined proportions. The model evaluates these mixed samples to determine their confidence levels. Finally, we rank samples by confidence scores and select data indices with the lowest-confidence values, adding the corresponding original samples to the labeled candidate set to complete each selection round.

### 3.1. Formal Definition

Given an unlabeled data pool *U* and an initially empty labeled data pool *L*, the objective of active learning is to select a subset of samples $X_{i}$ from *U* based on a predefined annotation budget (e.g., selecting 1000 samples at a time from a pool of 50,000 samples). These selected samples are annotated by human experts and added to *L*, such that $L\leftarrow L\cup X_{i},Y_{i}$, where $Y_{i}$ represents the newly acquired annotations. The selected samples are subsequently removed from the unlabeled pool: $U\leftarrow U\setminus X_{i}$.

A neural network model is defined as a function $f_{\theta }$, where θ denotes the model parameters. For input data $U=X_{1},X_{2},\ldots ,X_{n}$, the model generates predictions $\hat{Y}i=f\theta \left(X_{i}\right)$. The model is trained by minimizing the cross-entropy loss function:

(1) $$\ell \left(Y,\hat{Y}\right)=-\frac{1}{N}\sum_{i=1}^{N}\sum_{c=1}^{C}y_{i,c}\log\left({\hat{y}}_{i,c}\right),$$

where *N* represents the number of samples, *C* denotes the number of classes, and ${\hat{y}}_{i,c}$ is the predicted probability that the *i*-th sample belongs to class *c*. The optimization objective is to minimize this loss function:

(2) $$\theta^{*}=\arg\min_{\theta }\ell \left(Y,f_{\theta }\left(X\right)\right).$$

In subsequent data selection phases, the trained model evaluates samples from the unlabeled pool *U* to obtain confidence scores for each instance. Based on these confidence estimates, the method selects samples to be added to the labeled pool *L*, initiating the next cycle of training and data selection.

### 3.2. Feature Extraction

The feature representation from the final layer of a convolutional neural network (CNN) captures the highest-level, most abstract features from input images. These features effectively encapsulate global information and complex patterns, making them particularly valuable for tasks such as classification and recognition.

For each sample in the unlabeled data pool $U=X_{1},X_{2},\ldots ,X_{n}$, we extract features from the last convolutional layer of the CNN. The output features are denoted as $F_{\mathrm{Conv}}\left(X_{i}\right)\in R^{d}$, where *d* represents the feature dimension. Here, $U_{i}$ denotes the feature representation of the *i*-th sample extracted through this layer. The feature extraction process is formalized as

(3) $$U_{i}=F_{\mathrm{Conv}}\left(X_{i}\right).$$

### 3.3. Distance Measured

Euclidean distance effectively captures geometric relationships in continuous feature spaces, making it well-suited for detecting subtle differences between samples and demonstrating high sensitivity to small variations in data. In contrast, Manhattan distance is particularly effective for measuring differences in discrete or sparse feature spaces.

To ensure fair comparisons and prevent magnitude differences from skewing similarity, we first normalize feature vectors. Let $U_{i}$ and $U_{j}$ denote the original feature vectors of samples *i* and *j*, respectively. We define their normalized counterparts as

$${\tilde{U}}_{i}:=\frac{U_{i}}{{∥U_{i}∥}_{2}},\;{\tilde{U}}_{j}:=\frac{U_{j}}{{∥U_{j}∥}_{2}},$$

where ${∥\cdot ∥}_{2}$ denotes the Euclidean (L2) norm.

Because Euclidean and Manhattan distances have different scales, directly averaging them can introduce bias. We therefore compute each distance on the normalized vectors and additionally normalize each distance by the feature dimension *d* to align scales before aggregation. The (per-dimension) Euclidean and Manhattan distances are then defined as

(4) $$d_{E}\left({\tilde{U}}_{i},{\tilde{U}}_{j}\right)=\frac{1}{\sqrt{d}}\;\sqrt{\sum_{k=1}^{d}{\left({\tilde{U}}_{ik}-{\tilde{U}}_{jk}\right)}^{2}},$$

(5) $$d_{M}\left({\tilde{U}}_{i},{\tilde{U}}_{j}\right)=\frac{1}{d}\;\sum_{k=1}^{d}\left|{\tilde{U}}_{ik}-{\tilde{U}}_{jk}\right|.$$

The combined distance metric, obtained by averaging the normalized distances in Equations (4) and (5), is expressed as

(6) $$d_{c}\left(U_{i},U_{j}\right)=\frac{d_{E}\left({\tilde{U}}_{i},{\tilde{U}}_{j}\right)+d_{M}\left({\tilde{U}}_{i},{\tilde{U}}_{j}\right)}{2}.$$

### 3.4. Linear Data Mixing

The distance function $d_{c}\left(U_{i},U_{j}\right)$ calculates the similarity between feature samples $U_{i}$ and $U_{j}$. In this step, we select *n* samples $X_{1},X_{2},\ldots ,X_{n}$ that are most similar to sample $X_{i}$ for linear mixing. We denote the set of nearest neighbors of $U_{i}$ based on $d_{c}\left(U_{i},U_{j}\right)$ as $N_{i}$, and employ the parameter λ to control the degree of mixing. This process operates directly at the data level rather than on feature representations. The mixing formula is expressed as

(7) $$\hat{X_{i}}=\lambda \cdot X_{i}+\left(1-\lambda \right)\cdot \sum_{j\in N_{i}}X_{j},$$

where ${\hat{X}}_{i}$ represents the mixed data sample derived from $X_{i}$, $N_{i}$ denotes the index set of the *n* nearest neighbors of sample $X_{i}$, and λ controls the mixing weight of the original sample $X_{i}$. The linearly mixed sample ${\hat{X}}_{i}$ is then fed into the pre-trained model for prediction:

(8) $$P_{i}=f_{\theta }\left({\hat{X}}_{i}\right).$$

The output of the classification model, $P_{i}$, is typically a vector representing the predicted logits for each class. This output is converted into a probability distribution using the softmax function:

(9) $$\mathrm{Softmax}\left(P_{i}\right)=\left[\frac{\exp(P_{i1})}{\sum_{c}\exp\left(P_{ic}\right)},\ldots ,\frac{\exp(P_{iC})}{\sum_{c}\exp\left(P_{ic}\right)}\right],$$

where *C* represents the number of classes and $P_{ic}$ denotes the model’s logit score for sample ${\hat{X}}_{i}$ belonging to class *c*.

The probability distribution from the classification model’s output is utilized to determine the confidence level for each sample. The highest predicted probability typically serves as a confidence indicator:

(10) $$C_{i}=\max\left(\mathrm{softmax}\left(P_{i}\right)\right).$$

This represents the model’s maximum predicted probability for sample ${\hat{X}}_{i}$, indicating its confidence level. Samples are ranked by their confidence scores, and those with the lowest confidence are selected for the next active learning batch. When confidence levels are sorted in ascending order, sort[0] corresponds to the index of the sample with the lowest confidence, while sort[N−1] represents the highest confidence sample. From this sorted arrangement, we select the *n* samples with the lowest confidence scores for annotation. The indices of these selected samples constitute the active learning dataset $D_{a}$:

(11) $$D_{a}=\left\{X_{\mathrm{sort}[0]},X_{\mathrm{sort}[1]},\ldots ,X_{\mathrm{sort}[N-1]}\right\}.$$

We return the selected *n* sample indices for active learning annotation. The corresponding samples are retrieved from the original unlabeled dataset $U=X_{1},X_{2},\ldots ,X_{m}$ using these indices and added to the labeled pool *L*. This process is repeated iteratively throughout the entire active learning cycle, as shown in Algorithm 1.
**Algorithm 1** Distance-Measured Data Mixing Active Learning (DM2)  1:**Input:** $f_{\theta }$: randomly initialized neural network, *U*: unlabeled data pool,  2:        *L*: initial labeled data pool, *B*: query budget per round,  3:        *T*: number of acquisition rounds, *k*: number of neighbors, λ: MixUp parameter  4:**Output:** *L*: updated labeled pool  5:**Begin:**  6:        **for** round←1 to *T* **do**  7:               Train the model $f_{\theta }$ on the current labeled pool *L*.  8:               Extract features $\left\{F_{i}=F_{\mathrm{conv}}\left(x_{i}\right)\right\}$ for all samples $\{x_{i}\}\in U$.  9:               **for** each sample $x_{i}\in U$ **do**10:                        Compute distance $\delta_{ij}=\left|\right|F_{i}-F_{j}{\left|\right|}_{1}$ to all other samples $x_{j}\in U$.11:                        Identify $N(x_{i})$, the set of *k*-nearest neighbors to $x_{i}$ based on $\delta_{ij}$.12:               **end for**13:               Initialize an empty set for acquisition scores, S←{}.14:               **for** each sample $x_{i}\in U$ **do**15:                        Randomly select one neighbor $x_{j}$ from its neighbor set $N(x_{i})$.16:                        Generate a synthetic sample: ${\hat{x}}_{i}=\lambda x_{i}+\left(1-\lambda \right)x_{j}$.17:                        Calculate model output probabilities for the synthetic sample: $p\left(\hat{y}|{\hat{x}}_{i}\right)=\mathrm{Softmax}\left(f_{\theta }\left({\hat{x}}_{i}\right)\right)$.18:                        Compute the uncertainty score $S_{i}=\mathrm{Entropy}\left(p\left(\hat{y}|{\hat{x}}_{i}\right)\right)$.19:               **end for**20:               Select a set of *B* samples $X_{B}=\left\{x_{i}\in U\right\}$ corresponding to the highest scores in *S*.21:               Query the true labels $Y_{B}$ for the selected samples in $X_{B}$.22:               Add the newly labeled data to the labeled pool: $L\leftarrow L\cup (X_{B},Y_{B})$.23:               Remove the selected samples from the unlabeled pool: $U\leftarrow U\setminus X_{B}$.24:        **end for**25:**Return** *L*

## 4. Adversarial Training for Boundary Data Feature Fusion

This section presents an active learning algorithm that integrates adversarial training with feature fusion for boundary data samples. This method employs adversarial training to enhance model robustness, thereby strengthening performance when processing noisy and complex data. Simultaneously, the active learning strategy reduces the required number of training samples, lowering overall training costs. Specifically, in our boundary data feature fusion approach for active learning, samples selected in each round are initially augmented through adversarial training to generate adversarial counterparts. These adversarial samples are subsequently merged with the existing labeled pool, enabling the model to fully exploit the augmented data during updates.

**Figure 3:**
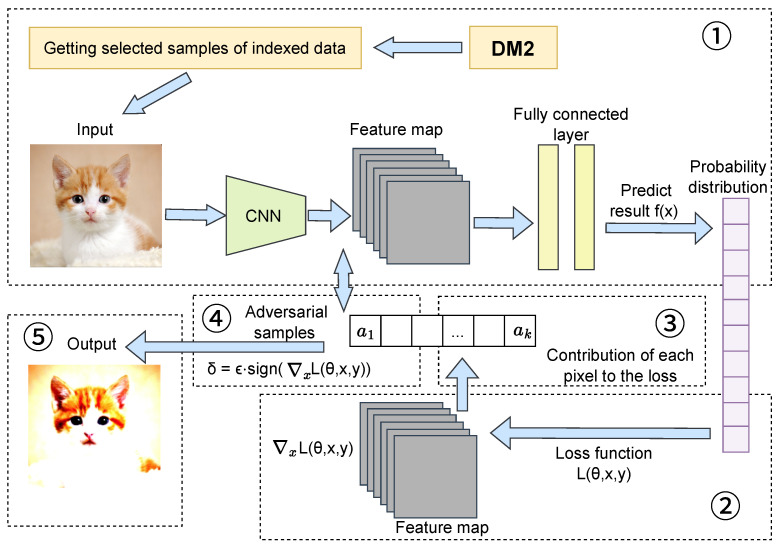
Overview of adversarial training for sample selection, where the input consists of near-boundary data selected by DM2 from Section 3. After generating adversarial samples, the original samples are added to the labeling pool alongside their adversarial counterparts. Through iterative selection of both original and adversarial sample sets, the model’s robustness is enhanced.

The advantage of this approach lies in its incorporation of active learning properties to reduce labeled data requirements while leveraging adversarial training to enhance the model’s classification capabilities, particularly when confronting noise and interference in real-world scenarios. Through this combination, the model’s information recognition performance is significantly improved, achieving more accurate classification in complex environments and demonstrating adaptability across diverse application scenarios. This algorithm not only improves model stability and classification accuracy but also reduces training sample requirements while adapting to large-scale dataset challenges, offering substantial practical value, especially in applications requiring rapid deployment and efficient training. The complete methodological process is illustrated in Figure 3.

### 4.1. FGSM Confrontation Training

Adversarial training serves as a method to improve model generalization by incorporating adversarial samples into the training dataset. This approach compels the model to learn from these challenging examples, thereby improving its ability to defend against adversarial perturbations. The Fast Gradient Sign Method (FGSM) represents one of the most widely used techniques to generate adversarial samples, and FGSM adversarial training constitutes a training methodology that employs FGSM to generate adversarial samples and incorporate them into the training set [33].

FGSM is an algorithm that efficiently generates adversarial perturbations by computing the gradient of the loss function with respect to the input. The fundamental principle involves applying a small perturbation along the direction of the loss function’s gradient to input samples, thereby causing the model to produce erroneous predictions. This perturbation is computed individually for each input sample, making it inherently sample-specific [34]. The FGSM generation process follows these steps:

**Calculate the gradient:** For each input sample and its corresponding label, we first compute the gradient of the loss function with respect to the input:

(12) $$\nabla_{x}L\left(\theta ,x,y\right),$$

where L(θ,x,y) represents the loss function with model parameters θ, input sample *x*, and true label *y*. This gradient indicates the direction in which small changes to the input would most significantly increase the loss.

**Generate adversarial perturbations:** Using the computed gradient to generate adversarial perturbations, the key principle of FGSM involves computing the sign of the gradient (representing the direction of the gradient) and adding perturbations along that direction. The perturbation magnitude is controlled by a small constant parameter:

(13) $${\mathrm{adv}}_{\mathrm{sample}}=x+\epsilon \cdot \mathrm{sign}\left(\nabla_{x}L\left(\theta ,x,y\right)\right),$$

where sign(·) is the sign function that extracts the sign of each element in the gradient vector, and ϵ is the hyperparameter that controls the perturbation magnitude. This formula represents the process of applying a small perturbation to input samples along the direction of the loss function’s gradient. The sign function ensures that the perturbation moves in the direction that would maximally increase the loss, while the ϵ parameter bounds the perturbation size to maintain the adversarial sample’s similarity to the original input.

The generated adversarial samples are incorporated alongside the original samples during the training process, enabling the model to learn correct predictions when confronted with adversarially perturbed inputs. This approach enhances the model’s robustness by exposing it to challenging examples that lie near the decision boundary.

In adversarial training, the training process comprises two complementary components. First, positive sample training follows the traditional approach by utilizing original data for model training. Second, adversarial sample training incorporates adversarial samples generated using FGSM into the training data. During each training step, a batch of data is selected from the training set, where each sample consists of an input *x* and its corresponding label *y*. FGSM is then applied to generate adversarial perturbations for each sample, producing the corresponding adversarial examples.

The training procedure calculates losses for both the original samples and their adversarial counterparts, combining these losses for backpropagation to update model parameters, where L(θ,x,y) represents the loss computed on the original sample, and $L(\theta ,{\mathrm{adv}}_{\mathrm{sample}},y)$ denotes the loss computed on the adversarial sample:

(14) $$L_{\mathrm{total}}=\frac{1}{2}\left(L\left(\theta ,x,y\right)+L\left(\theta ,{\mathrm{adv}}_{\mathrm{sample}},y\right)\right),$$

where this combined loss function ensures that the model simultaneously learns to make correct predictions on both normal and adversarially perturbed inputs.

For all hyperparameters, unless otherwise specified, we adopt the following settings in all experiments. **FGSM perturbation magnitude *ϵ*:** We search over ϵ∈1/255, 2/255, 4/255, 8/255 for image input (pixel range [0,1]) and report the results for the selected value in each experiment; the default is ϵ=8/255. **Loss mixing coefficient *λ*:** We weight clean and adversarial losses as Ltotal=(1−λ)L(θ,x,y)+λL(θ,advsample,y). We tune λ over the range λ∈0.3,0.5,0.7 and use λ=0.5 by default (corresponding to Equation (14)). **Number of neighbors *k*:** For modules that require neighbor retrieval (e.g., regularization of the consistency of k-NN or augmentation based on neighborhood, when applicable in our pipeline), we select *k* from 5,10,20 with a default of k=10.

FGSM adversarial training constitutes an effective method for improving model robustness. By generating adversarial samples and incorporating them into the training data, this approach enhances the model’s ability to adapt to input perturbations, enabling the model to maintain performance when confronted with adversarial examples during inference.

### 4.2. Adversarial Training for Sample Selection

This method combines active learning and adversarial training to enhance model stability and performance through the following process:

First, the trained model performs forward propagation to extract feature representations from the final layer for each sample, as expressed in Equation (3). We then select the most representative samples by computing pairwise similarity using a combined Manhattan and Euclidean distance metric, as shown in Equation (6). This combination leverages the strengths of both metrics to achieve stable similarity calculations, particularly for features with varying scales.

After identifying the *n* most similar samples through inter-sample similarity calculations, we merge them using the MixUp fusion method to generate new training instances that enhance model generalization, as shown in Equation (7). The model evaluates classification confidence and returns the corresponding index list for active learning selection.

Following sample selection by the boundary data feature fusion algorithm, the original images undergo forward propagation through the neural network to obtain prediction results via the fully connected layer. FGSM then calculates the gradient of the loss function with respect to the input image through backpropagation, revealing how each pixel should be modified to maximize the loss. Larger gradient magnitudes indicate greater pixel impact on the loss function.

The perturbation is computed and adversarial samples are generated according to

(15) $$\delta =\epsilon \cdot \mathrm{sign}(\nabla_{x}L\left(\theta ,x,y\right)),$$

where ϵ represents the perturbation step size, $\nabla_{x}L\left(\theta ,x,y\right)$ denotes the gradient of the loss function with respect to input sample *x*, L(θ,x,y) is the model’s loss function, and sign(·) is the sign function. The parameter ϵ determines the perturbation magnitude: smaller values (e.g., ϵ=0.03) are used for simpler tasks like MNIST or SVHN, while larger values (e.g., ϵ=0.1) are selected for complex tasks like CIFAR-10. Larger perturbations make training for robust performance more challenging.

The calculated perturbations are added to the original samples to generate adversarial samples:

(16) H=x+δ,

where *H* represents the adversarial sample resulting from adding the perturbation δ to the original input *x*.

**Algorithm overview:** We work with a model $f_{\theta }$ that iteratively improves using an unlabeled pool *U* and a labeled set *L*. For each x∈U, we extract a feature embedding $F_{\mathrm{conv}}\left(x\right)\in R^{d}$ from the model’s penultimate layer and measure pairwise similarity using the combined distance $D\left(x_{i},x_{j}\right)={∥F_{\mathrm{conv}}\left(x_{i}\right)-F_{\mathrm{conv}}\left(x_{j}\right)∥}_{1}+{∥F_{\mathrm{conv}}\left(x_{i}\right)-F_{\mathrm{conv}}\left(x_{j}\right)∥}_{2}$. Each example *x* has a neighborhood N(x) consisting of its top-*n* most similar peers under *D*. We synthesize interpolated examples $\hat{x}=\lambda x+\left(1-\lambda \right)x^{\prime }$ using MixUp with λ∼Beta(α,α) and $x^{\prime }\in N\left(x\right)$ to probe the decision boundary and calibrate uncertainty.

Based on uncertainty $u(x;f_{\theta })$, we select the *B* most uncertain samples to form batch $X_{B}$ for labeling, obtaining labels $Y_{B}$. For each selected sample $x\in X_{B}$, we create an adversarial counterpart using FGSM: we form a pseudo-label $y^{\prime }\left(x\right)$ from the model’s current prediction, compute the input gradient $g\left(x\right)=\nabla_{x}L\left(f_{\theta }\left(x\right),y^{\prime }\left(x\right)\right)$, and craft a perturbation δ(x)=ε,sign(g(x)), producing the adversarial example H(x)=x+δ(x) (clipped to the valid input range). The labeled set is augmented with both clean and adversarial pairs (x,y),(H(x),y) for $x\in X_{B},y\in Y_{B}$.

Training minimizes the combined objective Ltotal=(1−λadv)Lorig+λadvLadv, where λadv balances clean accuracy and robustness, and ε controls perturbation strength. This cycle—feature extraction, neighborhood identification, uncertainty-based selection, and adversarial augmentation—repeats until convergence. By selecting samples that are uncertain and lie in dense feature regions, then training on their adversarial variants, the algorithm enhances model robustness while reducing labeling costs, as shown in Algorithm 2.
**Algorithm 2** Distance-Measured Data Mixing with Adversarial Training (DM2-AT)  1:**Input:** Model $f_{\theta }$, unlabeled pool *U*, labeled pool *L*, batch size *B*, neighbor count *n*, MixUp parameter α, FGSM step size ϵ  2:**Output:** Trained model $f_{\theta }$  3:**Begin:**  4:        **while** model has not converged **do**  5:                Extract features $F_{\mathrm{conv}}\left(x_{i}\right)$ for all $x_{i}\in U$.  6:                For each $x_{i}\in U$, find its top-*n* neighbors $N(x_{i})$ using L1+L2 distance.  7:                Generate synthetic set $\hat{X}$ via MixUp on pairs from *U* and their neighbors.  8:                Score $\hat{X}$ with model uncertainty to select the *B* most uncertain original samples $X_{B}\subset U$.  9:                **for** each $x_{i}\in X_{B}$ **do**10:                        $g_{i}\leftarrow \nabla_{x}L\left(f_{\theta }\left(x_{i}\right),y_{i}^{\prime }\right)$*// $y_{i}^{\prime }$ is the model’s predicted label*11:                        $H_{i}\leftarrow x_{i}+\epsilon \cdot \mathrm{sign}\left(g_{i}\right)$12:                **end for**13:                Query true labels $Y_{B}$ for the selected samples $X_{B}$.14:                Update $L\leftarrow L\cup \{\left(X_{B},Y_{B}\right),\left(H_{B},Y_{B}\right)\}$ and $U\leftarrow U\setminus X_{B}$.15:                Retrain $f_{\theta }$ on *L* using a combined loss for original and adversarial samples.16:        **end while**17:**Return** $f_{\theta }$

## 5. Theoretical Analysis

### 5.1. Notation and Setup

Let $U=\{X_{1},\ldots ,X_{m}\}$ denote the unlabeled pool and *L* the labeled pool. A model $f_{\theta }$ with parameters θ produces class probabilities ${\hat{Y}}_{i}=f_{\theta }\left(X_{i}\right)$ and is trained by minimizing the cross-entropy loss in (1), with optimal parameters $\theta^{*}$ given by (2). Feature embeddings are extracted by $U_{i}=F_{\mathrm{Conv}}\left(X_{i}\right)\in R^{d}$ as in (3). Distances are measured by $d_{E}$ and $d_{M}$ in (4)–(5) and combined as $d_{c}$ in (6). For each anchor $X_{i}$, a neighbor set $N_{i}$ is defined as the indices of the *n* nearest neighbors under $d_{c}$. Mixed inputs are formed at the data level by

(17) $${\hat{X}}_{i}=\lambda X_{i}+\left(1-\lambda \right)\sum_{j\in N_{i}}X_{j}\;\mathrm{with}\;\lambda \in \left[0,1\right],$$

as in (7). The model outputs logits $P_{i}=f_{\theta }\left({\hat{X}}_{i}\right)$, which are mapped to probabilities via softmax (9), and the confidence is $C_{i}=\max\left(\mathrm{softmax}\left(P_{i}\right)\right)$ in (10). The acquisition set comprises the indices of the *n* lowest-confidence samples, cf. (11).

### 5.2. Geometric Rationale for Distance-Weighted Mixing

We analyze the effect of DM2 on two axes crucial for active learning: (i) uncertainty exposure at decision boundaries; (ii) diversity preservation through local neighborhood mixing.

We work under standard conditions often met in deep representation spaces: (A1) The embedding $F_{\mathrm{Conv}}$ is locally Lipschitz: $∥F_{\mathrm{Conv}}\left(X\right)-F_{\mathrm{Conv}}\left(X^{\prime }\right)∥\leq L∥X-X^{\prime }∥$ for some L>0. (A2) The classifier head of $f_{\theta }$ is $L_{f}$-Lipschitz in input space on compact domains. (A3) Nearby points under $d_{c}$ have high label-correlation: there exists η∈[0,1) such that for $j\in N_{i}$, $P[Y_{j}\neq Y_{i}]\leq \eta$; equivalently, neighborhoods are label-homogeneous with bounded noise. (A4) Calibration around the decision boundary: near regions where class posteriors are close (small margin), confidence $\max_{c}{\hat{y}}_{ic}$ decreases monotonically with the distance to the margin hyper-surface.

Assumption (A1)–(A3) capture that $d_{c}$ is a surrogate for semantic proximity, while (A4) links geometric proximity to predictive uncertainty.

Consider a first-order expansion of $f_{\theta }$ w.r.t. the input:

(18) $$f_{\theta }\left({\hat{X}}_{i}\right)\approx f_{\theta }\left(\lambda X_{i}+\left(1-\lambda \right)\sum_{j\in N_{i}}X_{j}\right)\approx \lambda f_{\theta }\left(X_{i}\right)+\left(1-\lambda \right)\sum_{j\in N_{i}}f_{\theta }\left(X_{j}\right)+\varepsilon_{i},$$

with a remainder term $∥\varepsilon_{i}∥\leq \frac{1}{2}L_{f}{∥\lambda X_{i}+\left(1-\lambda \right)\sum_{j\in N_{i}}X_{j}-X_{i}∥}^{2}$ by (A2). Thus, to first order, the logits on the mixed input approximate an average of neighbor logits. When $N_{i}$ is label-homogeneous, the average logit sharpens the predicted class; when $N_{i}$ straddles a class boundary, the average logit becomes ambiguous, lowering confidence.

### 5.3. Uncertainty Amplification Near Class Boundaries

Define the pointwise margin for logits P(x) as

(19) $$\gamma \left(x\right)≜P_{\left(1\right)}\left(x\right)-P_{\left(2\right)}\left(x\right),$$

the gap between the top-two logits. By softmax monotonicity, smaller γ(x) implies lower confidence C(x).

**Lemma** **1.** *Margin reduction under heterogeneous neighborhoods. Let $X_{i}$ have neighbors $N_{i}$ with class proportions $\pi_{c}$ ($\sum_{c}\pi_{c}=1$), and suppose $f_{\theta }$ is locally linear around $\left\{X_{i}\right\}\cup \left\{X_{j}:j\in N_{i}\right\}$. Then, for the mixed input ${\hat{X}}_{i}$ with weight λ∈(0,1),*

(20) $$\gamma \left({\hat{X}}_{i}\right)\approx \lambda \;\gamma \left(X_{i}\right)+\left(1-\lambda \right)\;\Delta_{i},$$

*where $\Delta_{i}$ is the top-two logit gap of the neighbor-averaged prediction ${\bar{P}}_{i}=\sum_{j\in N_{i}}P\left(X_{j}\right)$. If $N_{i}$ spans multiple classes so that ${\bar{P}}_{i}$ is class-ambiguous, then $\Delta_{i}$ is small, and hence $\gamma \left({\hat{X}}_{i}\right)\leq \lambda \;\gamma \left(X_{i}\right)$, yielding $C_{i}$ reduced relative to $C(X_{i})$.*

**Proof.**  Local linearity yields $P\left({\hat{X}}_{i}\right)\approx \lambda P\left(X_{i}\right)+\left(1-\lambda \right){\bar{P}}_{i}$. Let a(·) denote the top-two logit gap (an affine functional restricted to the two dominant coordinates). Then $a\left(\lambda p+\left(1-\lambda \right)\bar{p}\right)=\lambda a\left(p\right)+\left(1-\lambda \right)a\left(\bar{p}\right)$ for any logits $p,\bar{p}$ sharing the same top-two ordering; otherwise, the gap cannot increase beyond the convex combination by triangle inequality. Hence, $\gamma \left({\hat{X}}_{i}\right)\leq \lambda \gamma \left(X_{i}\right)+\left(1-\lambda \right)\Delta_{i}$. If neighbors are heterogeneous, $\Delta_{i}$ is small due to averaging conflicting logits, which lowers $\gamma ({\hat{X}}_{i})$ and therefore $C_{i}$ by softmax monotonicity in the gap. □

Samples whose neighbor sets $N_{i}$ cross decision boundaries are systematically assigned lower confidence after mixing and are thus prioritized by DM2. This aligns selection with true boundary regions where labels are most informative for reducing model uncertainty.

### 5.4. Diversity Preservation via Distance Coupling

Let G be the *k*-NN graph on $\left\{U_{i}\right\}$ under $d_{c}$. DM2 forms mixes anchored at many distinct nodes with their local neighborhoods. If the acquisition selects the *n* lowest-confidence anchors after mixing, these anchors tend to be located on edges or cuts of G that cross clusters. Under mild clusterability:

(A5) The embedding decomposes into *r* well-separated clusters $\left\{C_{1},\ldots ,C_{r}\right\}$ with inter-cluster distances larger than intra-cluster distances under $d_{c}$.

Then, boundary regions appear around each cut $\left(C_{a},C_{b}\right)$; mixed inputs that pool neighbors from both $C_{a}$ and $C_{b}$ reduce confidence within each cut. Consequently, the *n* lowest-confidence anchors are spread across multiple cuts, promoting diversity without explicit diversity regularizers.

### 5.5. Stability of Mixed Confidence Under Neighbor Noise

Consider neighbor noise: a fraction ρ of $N_{i}$ are erroneous neighbors (e.g., misembedded or outliers). Let $P_{i}^{\mathrm{true}}$ be the average logits over true semantic neighbors and $P_{i}^{\mathrm{noise}}$ over noisy neighbors. Then

(21) $$P\left({\hat{X}}_{i}\right)\approx \lambda P\left(X_{i}\right)+\left(1-\lambda \right)\left(\left(1-\rho \right)P_{i}^{\mathrm{true}}+\rho P_{i}^{\mathrm{noise}}\right).$$

If $∥P_{i}^{\mathrm{noise}}-P_{i}^{\mathrm{true}}∥\leq \delta$ (bounded contamination), the perturbation to logits is at most (1−λ)ρδ, so the induced confidence change satisfies

(22) $$|C\left({\hat{X}}_{i}\right)-\tilde{C}\left({\hat{X}}_{i}\right)|\leq L_{\mathrm{sm}}\left(1-\lambda \right)\rho \delta ,$$

where $L_{\mathrm{sm}}$ is the Lipschitz constant of the softmax–max operator. Thus, DM2 confidence is robust to small neighbor noise for moderate λ.

### 5.6. Choice of the Combined Distance $d_{c}$

The combined metric $d_{c}=\frac{1}{2}\left(d_{E}+d_{M}\right)$ inherits the following:

(i) Metric property: Since $d_{E}$ and $d_{M}$ are metrics on $R^{d}$, any positive weighted sum is a metric. Hence $d_{c}$ satisfies non-negativity, symmetry, and the triangle inequality.

(ii) Sensitivity balance: $d_{E}$ is sensitive to dense directions, while $d_{M}$ is robust to sparse, axis-aligned deviations. Averaging thus mitigates anisotropy and promotes stable neighbor sets in heterogeneous embeddings.

$d_{c}$ defined by (6) is a metric on $R^{d}$.

**Proof.**  For all $x,y,z\in R^{d}$: Non-negativity and identity of indiscernibles follow from those of $d_{E}$ and $d_{M}$. Symmetry is immediate. For the triangle inequality,

(23) $$\begin{array}{cc} d_{c}\left(x,z\right) & =\frac{1}{2}\left(d_{E}\left(x,z\right)+d_{M}\left(x,z\right)\right)\\ & \leq \frac{1}{2}\left(d_{E}\left(x,y\right)+d_{E}\left(y,z\right)+d_{M}\left(x,y\right)+d_{M}\left(y,z\right)\right)\\ & =d_{c}\left(x,y\right)+d_{c}\left(y,z\right). \end{array}$$

□

### 5.7. Acquisition Optimality Under a Localized Fisher Criterion

Let Σ(x) denote the conditional Fisher information of $f_{\theta }$ at input *x* with respect to parameters θ (under the model distribution). For classification with softmax outputs, points near the decision boundary tend to have larger Fisher trace trΣ(x), which correlates with higher expected gradient magnitude.

Define the mixed-point Fisher score

(24) $$\Phi_{i}\left(\lambda \right)≜E\left[∥\nabla_{\theta }\ell \left(Y,f_{\theta }\left({\hat{X}}_{i}\right)\right){∥}^{2}\;|\;X_{i},N_{i}\right]\propto \mathrm{tr}\;\Sigma \left({\hat{X}}_{i}\right).$$

Under (A1)–(A4) and local linearization, if $N_{i}$ is heterogeneous, ${\hat{X}}_{i}$ approaches the boundary and $\mathrm{tr}\;\Sigma ({\hat{X}}_{i})$ increases. Therefore selecting minimum-confidence ${\hat{X}}_{i}$ approximately maximizes $\Phi_{i}\left(\lambda \right)$ among anchors, aligning DM2 with a proxy of information gain.

**Theorem** **1.** *Informative selection under DM2. Suppose (A1)–(A5) hold and that $f_{\theta }$ is locally linear in a neighborhood containing $\left\{X_{i}\right\}\cup \left\{X_{j}:j\in N_{i}\right\}$. Then, for any fixed λ∈(0,1), ranking anchors $X_{i}$ by ascending confidence $C_{i}$ on mixed inputs ${\hat{X}}_{i}$ is equivalent to ranking by a non-increasing function of the margin $\gamma ({\hat{X}}_{i})$ and thus, up to a monotone transform, by $\mathrm{tr}\;\Sigma ({\hat{X}}_{i})$. Consequently, the DM2 acquisition set approximates a maximizer of the localized Fisher score among anchors, favoring boundary-spanning, diverse regions of the data manifold.*

**Proof sketch.**  Softmax confidence is a monotone function of the logit gap $\gamma ({\hat{X}}_{i})$; hence, ordering by $C_{i}$ equals ordering by $\gamma ({\hat{X}}_{i})$. Under local linearization and (A4), smaller $\gamma ({\hat{X}}_{i})$ implies proximity to the decision boundary, where the Fisher information increases for multinomial logistic models. Thus, ranking by $C_{i}$ approximates ranking by $\mathrm{tr}\;\Sigma ({\hat{X}}_{i})$. Cluster separation (A5) ensures that anchors selected across different cuts yield coverage of multiple boundary regions (diversity). □

### 5.8. On the Mixing Coefficient λ

The coefficient λ trades off anchor faithfulness and boundary probing.

If λ→1, ${\hat{X}}_{i}\to X_{i}$, recovering standard uncertainty sampling. If λ→0, ${\hat{X}}_{i}$ collapses to neighbor averages, which may over-smooth and obscure fine boundaries. Under (A3), there exists an interval Λ⊂(0,1) such that for λ∈Λ, heterogeneous neighborhoods strictly reduce $\gamma ({\hat{X}}_{i})$ relative to $\gamma (X_{i})$ while homogeneous neighborhoods preserve or increase it. Therefore, DM2 self-selects anchors with heterogeneous $N_{i}$.

Existence of a beneficial mixing range. Assume there exist anchors with $\gamma (X_{i})>0$ and heterogeneous $N_{i}$ such that $\Delta_{i}<\gamma \left(X_{i}\right)$ in Lemma 1. Then, for any λ∈(0,1),

(25) $$\gamma \left({\hat{X}}_{i}\right)\leq \lambda \;\gamma \left(X_{i}\right)+\left(1-\lambda \right)\Delta_{i}<\gamma \left(X_{i}\right).$$

Thus, confidence strictly decreases for such anchors; conversely, if $N_{i}$ is homogeneous with large margin, confidence is non-decreasing for λ near 1.

**Proof.**  Immediate from Lemma 1 and the strict inequality $\Delta_{i}<\gamma \left(X_{i}\right)$. □

### 5.9. Considerations and Summary

Let m=|U| and *d* be the feature dimension. Computing pairwise $d_{c}$ naively is $O(m^{2}d)$; approximate *k*-NN reduces this to near-linear time in *m*. Mixing and forward passes scale as $O(mn\cdot C_{f})$, where $n=|N_{i}|$ and $C_{f}$ is model inference cost. Hence, with approximate neighbors and mini-batched evaluation, DM2 scales to large pools.

Mixing within $d_{c}$-based neighborhoods yields mixed inputs whose logits approximate convex combinations of neighbor logits. Heterogeneous neighborhoods reduce the logit margin and thus confidence, preferentially surfacing boundary samples for labeling. The acquisition is robust-to-moderate neighbor noise and approximates selection by localized Fisher information. The combined distance $d_{c}$ is a proper metric that balances Euclidean and Manhattan sensitivities, stabilizing neighbor selection.

## 6. Experimental Results

We evaluate our method against state-of-the-art and baseline active learning approaches, including random selection, least confidence selection, and entropy sampling. Our approach is validated on both balanced and imbalanced image classification tasks using MobileNet [21] architectures. Experiments employ 7 to 10 active learning cycles with labeling budgets ranging from 20 to over 2500 samples per cycle. Data selections follow standard active learning practices without replacement, and all results are averaged over 5 runs. All experiments are implemented using PyTorch 2.2 [35].

For MNIST [36], we employ a CNN classification model with the Adam optimizer [37] at learning rate $10^{-3}$ and batch size 96, training for 50 epochs per cycle. For CIFAR-10, CIFAR-10s [38], CIFAR-10C [39], and SVHN [40], we use MobileNet [21] with the SGD optimizer [41], initial learning rate 0.1, batch size 128, momentum 0.9, and weight decay $5\times 10^{-4}$. Training proceeds for 200 epochs with learning rate decay to 0.01 at epoch 160. We compare against established baselines including Random Selection [3], Entropy [22], Least Confidence [5], Margin [6], BALD [4], CoreSet [12], EntropyBayesian [7], UncertainGCN [42], BADGE [19], ProbCover [28], Alpha-Mix [1], and NoiseStability [32], using identical parameters for fair comparison.

Table 1 demonstrates that our method outperforms all other active learning approaches across most datasets. The DM2 method achieves superior performance compared to all baseline active learning methods on all datasets except MNIST. For the MNIST dataset, the simplicity and limited data volume result in the boundary-based selection model failing to learn useful information more rapidly than simpler selection strategies. The excellent performance on the SVHN dataset demonstrates that the DM2 method exhibits strong stability when handling imbalanced datasets. This robustness likely stems from the method’s design, which avoids data imbalance issues that can negatively impact auxiliary model training and subsequently degrade task model performance. In experiments with CIFAR-10s containing noisy data, the DM2 method successfully identifies samples conducive to model learning and demonstrates superior noise stability compared to competing approaches.

To further validate our method’s robustness, we conducted additional experiments using CIFAR-10s and SVHN datasets with ResNet18 [43] and VGG16 [44] architectures. Standard data augmentation techniques were applied during training, including random horizontal flipping and cropping. As shown in Table 2, our method consistently outperforms all other active learning approaches across these more complex architectures.

### 6.1. Robustness for Adversarial Training

The comparative experimental results for different active learning strategies are presented in Table 3. The results demonstrate that our method incorporating adversarial training achieves higher model accuracy compared to other active learning approaches, indicating that the boundary data feature fusion algorithm with adversarial training proposed in this work effectively improves model performance.

The experiments also reveal that conventional active learning methods often struggle to enhance performance on test sets in complex environments. Traditional active learning approaches learn only from clean datasets and exhibit reduced effectiveness when recognizing data in challenging conditions. By integrating adversarial training with the method from Section 4, our approach generates adversarial samples based on selected data, enhancing the model’s ability to learn from difficult-to-classify samples. This enables more effective integration and learning from data in complex environments, thereby improving overall performance.

The experimental results in Table 3 show that the DM2-AT method proposed in this work significantly outperforms competing methods across different models and datasets, confirming the effectiveness of our approach. The method increases learning challenges by generating adversarial samples through adversarial training on selected samples. When facing real-world scenarios with complex environments, this approach demonstrates stronger capability in identifying samples under noise interference, efficiently reducing the data requirements for building machine learning models while achieving superior performance.

### 6.2. Convergence Analysis

**Figure 4:**
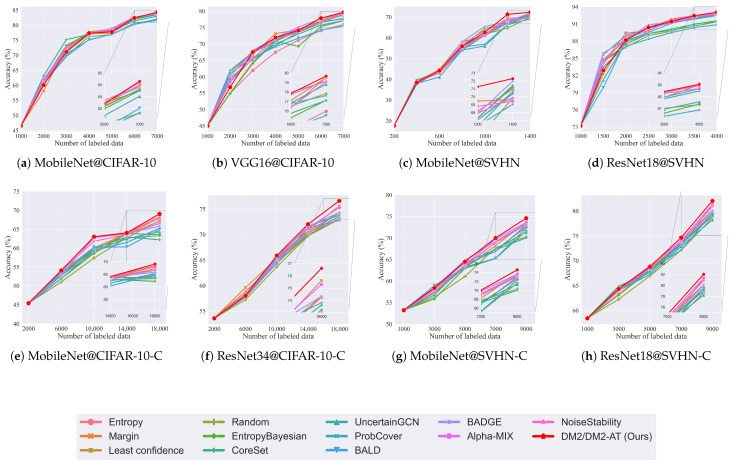
Classification performance of different methods across various datasets and models. The graphs are divided into two groups: experiments on datasets for DM2 are shown at the upper panels, while experiments for DM2-AT are at the lower panels. Our method shows significant advantages across multiple datasets and models, proving its wide applicability.

To analyze the performance trends of our method across different data selection cycles, we plotted convergence graphs based on the results from Table 1 and Table 2. Figure 4 presents trend plots for six different datasets and model combinations. All experimental values are averaged over three runs using consistent learning rates and parameters across all methods for fair comparison.

Figure 4 is organized into two groups: the upper panels show results for DM2, while the lower panels display results for DM2-AT. The method demonstrates gradual improvement across subsequent epochs, particularly in CIFAR-10 experiments using MobileNet and VGG16 architectures, where our approach surpasses competing methods. For CIFAR-10s, the model quickly learns to select more relevant data, ultimately outperforming all alternatives. On SVHN, our method demonstrates clear superiority by the 6th round, consistently outperforming other approaches throughout the remaining cycles.

Our method exhibits significant advantages across multiple datasets and model architectures, demonstrating broad applicability. In contrast, competing methods show less adaptability and inconsistent performance. Panel (e) reveals that the CIFAR-10C dataset presents challenges, with the trend chart showing some fluctuations in data selection performance. In panel (f), experiments using CIFAR-10C with ResNet34 show smoother progression and higher performance compared to MobileNet.

The ResNet18 model trained on SVHN-C in panel (h) exhibits favorable convergence trends. Except for the 3000th iteration where DM2-AT did not surpass several competing methods, it achieved excellent results across all other iterations. In panel (g), SVHN-C initially presents challenges during early training. However, after processing 5000 data points, the model rapidly identifies samples with stronger feature information, leading to sharp performance improvements and ultimately achieving superior results. These findings demonstrate the effectiveness of our active learning approach that fuses features from adversarial training boundary data.

### 6.3. Time Efficiency

The computational efficiency of active learning methods depends on the cost of sample distance calculations and subset selection procedures, as presented in Table 4. We evaluated the time efficiency of several effective methods using identical hyperparameters from our experiments. Our methods demonstrate computational efficiency comparable to state-of-the-art algorithms and exhibit favorable scalability as the annotation budget or number of categories increases.

### 6.4. Ablation Study

The active learning method proposed in this work incorporates two key components: boundary data feature fusion and adversarial training. Since removing adversarial training yields a method similar to that in Section 3, we conduct ablation experiments focusing on sample distances, fusion ratios, and perturbation values.

To assess method validity, we replace components in the ablation study: using Euclidean distance instead of the combined Euclidean and Manhattan distance, employing equal-weight fusion instead of adaptive fusion ratios, and using a fixed perturbation value of 0.05 instead of adaptive values. Table 5 presents the ablation results, demonstrating that adversarial training significantly enhances fault tolerance and robustness.

According to the experimental results in Table 5, replacing the combined distance metric with Euclidean distance alone struggles to accurately capture data distributions across different datasets, frequently leading to model confusion and suboptimal performance. Ablation experiments using equal-weight fusion show that uniform fusion causes merged features to lose distinctiveness, resulting in deteriorated model recognition performance.

The ablation study confirms that the adversarial training-based boundary data feature fusion algorithm enhances sample efficiency in active learning while improving the model’s generalization capability across diverse datasets and challenging conditions.

Active learning represents a prominent research direction for deep neural networks, enabling efficient model training with reduced sample requirements. We propose a simple yet stable method that exploits inter-sample relationships and data distribution characteristics. Through uncertainty prediction based on similarity measures and weighted mixing strategies, our approach demonstrates superior performance in both theoretical analysis and experimental evaluation across multiple tasks. The integration of adversarial training with boundary data feature fusion further enhances model robustness and generalization capability in complex environments.

Future work will focus on more challenging scenarios where the computational efficiency and cost reduction benefits of active learning become increasingly significant. We aim to extend our approach to handle larger-scale datasets and more complex domain adaptation problems, where traditional supervised learning approaches face substantial annotation costs and computational constraints. 

## Figures and Tables

**Table 1:** Accuracy (%) of experimental results with different datasets.

Methods	MNIST	CIFAR-10	CIFAR-10s	SVHN	Avg
Entropy [22] (IJCNN14)	92.78 ± 0.14	84.00 ± 0.13	64.00 ± 1.42	70.91 ± 9.01	77.92
Margin [6] (WIREs14)	**93.42 ± 0.13**	83.62 ± 0.07	63.47 ± 0.37	69.64 ± 4.84	77.53
Least Confidence [5]	92.98 ± 0.13	83.51 ± 0.04	63.89 ± 1.79	70.52 ± 2.25	77.73
Random [3]	87.78 ± 0.32	81.62 ± 0.19	62.94 ± 0.01	69.82 ± 3.42	75.54
EntropyBayesian [7] (ICML16)	92.05 ± 0.65	83.62 ± 0.25	61.82 ± 0.56	71.21 ± 4.13	77.18
CoreSet [12]	89.45 ± 1.12	83.04 ± 0.14	63.78 ± 0.72	71.41 ± 1.94	76.85
UncertainGCN [42] (NIPS20)	87.56 ± 2.47	83.51 ± 0.10	61.40 ± 4.43	70.65 ± 0.07	75.78
ProbCover [28] (NIPS22)	88.75 ± 0.68	81.63 ± 0.17	64.13 ± 1.88	69.04 ± 12.58	75.89
BALD [4] (ICML17)	92.55 ± 1.22	82.03 ± 0.22	63.62 ± 1.81	70.36 ± 3.12	77.14
BADGE [19]	92.35 ± 0.06	83.83 ± 0.05	62.46 ± 1.69	71.94 ± 2.35	77.65
Alpha-Mix [1] (CVPR22)	93.10 ± 0.46	84.22 ± 0.05	62.58 ± 0.72	69.43 ± 0.28	77.25
NoiseStability [32] (IJCAI24)	92.63 ± 0.37	83.87 ± 0.04	62.55 ± 0.36	69.87 ± 19.85	77.23
**DM2 (Ours)**	93.15 ± 0.32	**84.27 ± 0.01**	**64.71 ± 3.82**	**72.23 ± 2.41**	**78.59**

Bold denotes the best result and underline denotes the second result in each column.

**Table 2:** Accuracy (%) of experimental results under large scale models.

Methods	SVHN (ResNet18)	CIFAR-10 (vgg16)	Avg
Entropy [22]	92.94 ± 0.04	75.97 ± 5.56	84.46
Margin [6]	92.99 ± 0.01	79.51 ± 0.24	86.25
Least Confidence [5]	93.02 ± 0.01	79.57 ± 0.18	86.3
Random [3]	91.32 ± 0.09	77.84 ± 0.08	84.58
EntropyBayesian [7]	91.38 ± 0.09	77.12 ± 3.34	84.27
CoreSet [12]	92.48 ± 0.01	77.10 ± 0.08	84.79
UncertainGCN [42]	91.57 ± 0.06	78.82 ± 0.05	85.20
ProbCover [28]	90.88 ± 0.19	77.62 ± 0.17	84.25
BALD [4]	92.67 ± 0.02	75.51 ± 1.2	84.09
BADGE [19]	93.04 ± 0.06	79.20 ± 0.76	86.12
Alpha-Mix [1]	92.69 ± 0.03	79.06 ± 0.34	85.88
NoiseStability [32]	92.91 ± 0.04	79.10 ± 0.42	86.01
**DM2 (Ours)**	**93.04 ± 0.04**	**79.71 ± 0.09**	**86.38**

Bold denotes the best result in each column.

**Table 3:** Comparison of accuracy results of different methods on different models (%).

Methods	MNIST-C (CNN)	SVHN-C (MobileNet)	CIFAR10-C (MobileNet)	SVHN-C (ResNet18)	CIFAR10-C (ResNet34)
Entropy [22]	84.74 ± 2.54	72.51 ± 1.25	67.21 ± 0.54	79.53 ± 0.89	74.25 ± 2.56
Least Confidence [5]	85.62 ± 1.67	73.66 ± 0.72	68.35 ± 1.85	81.20 ± 2.56	75.61 ± 1.87
Margin [6]	84.83 ± 1.34	73.24 ± 1.54	67.04 ± 1.78	79.98 ± 2.28	74.32 ± 1.43
Random [3]	82.61 ± 3.78	70.28 ± 2.66	63.27 ± 0.28	78.61 ± 3.95	73.01 ± 1.22
EntropyBayesian [7]	83.34 ± 1.32	71.64 ± 2.84	63.59 ± 2.72	78.91 ± 0.31	73.20 ± 0.18
CoreSet [12]	84.52 ± 2.54	72.91 ± 1.52	62.18 ± 3.36	79.01 ± 1.02	73.61 ± 2.45
UncertainGCN [42]	83.11 ± 1.98	71.37 ± 1.73	64.36 ± 1.84	78.23 ± 2.43	72.93 ± 1.95
ProbCover [28]	80.13 ± 2.75	70.01 ± 3.43	65.23 ± 0.76	79.54 ± 3.61	73.81 ± 3.68
BALD [4]	84.55 ± 1.43	72.29 ± 2.85	64.87 ± 2.05	79.02 ± 0.88	72.77 ± 2.84
BADGE [19]	85.78 ± 0.74	73.35 ± 1.94	65.79 ± 1.96	80.11 ± 1.96	74.32 ± 1.29
Alpha-Mix [1]	85.91 ± 0.32	73.59 ± 1.22	67.91 ± 2.67	81.32 ± 1.61	75.29 ± 2.71
NoiseStability [32]	85.26 ± 1.22	73.03 ± 2.51	66.52 ± 1.43	80.89 ± 2.27	73.04 ± 1.43
DM2-AT (Ours)	**86.63 ± 0.91**	**74.56 ± 2.02**	**69.05 ± 1.77**	**82.02 ± 2.51**	**76.63 ± 1.82**

Bold denotes the best result in each column.

**Table 4:** Time effect result.

Method	Mnist (/s)	CIFAR-10 (/m)
BADGE	2	2.07
EntropyBayesian	4	3.25
BALD	16	4.58
NoiseStability	20	4.21
**Our**	3	**2.07**

Bold denotes the best result in each column.

**Table 5:** Ablation study: accuracy comparison results (%).

Datasets	-Distance	-Fusion Ratio	-Perturbation	DM2-AT
MNIST-C (CNN)	83.45 ± 0.97	84.29 ± 1.45	85.62 ± 0.51	**86.63 ± 0.91**
CIFAR10-C (MobileNet)	73.32 ± 1.44	72.24 ± 2.31	73.52 ± 1.32	**74.56 ± 2.02**
SVHN-C (MobileNet)	66.49 ± 1.41	66.22 ± 0.83	67.13 ± 1.42	**69.05 ± 1.77**
SVHN-C (ResNet18)	78.89 ± 1.53	80.35 ± 2.89	81.02 ± 0.84	**82.02 ± 2.51**
CIFAR10-C (ResNet34)	74.93 ± 0.71	75.44 ± 1.37	75.58 ± 2.32	**76.63 ± 1.82**

Bold denotes the best result in each column.

## Data Availability

The data presented in this study are openly available in [CIFAR-10] [https://www.cs.utoronto.ca/~kriz/learning-features-2009-TR.pdf] (accessed on 1 October 2025) [21].
